# Lichenoid tissue eruption of the vulva associated with immune checkpoint inhibitor

**DOI:** 10.1097/JW9.0000000000000161

**Published:** 2024-07-01

**Authors:** Maya I. Davis, David J. DiCaudo, Leah A. Swanson

**Affiliations:** a Mayo Clinic Alix School of Medicine, Scottsdale, Arizona; b Department of Dermatology, Mayo Clinic, Scottsdale, Arizona; c Department of Laboratory Medicine and Pathology, Mayo Clinic, Scottsdale, Arizona

**Keywords:** erosive lichen planus, vulvar dermatoses, immune checkpoint inhibitor, lichenoid tissue reaction

**What is known about this subject in regard to women and their families?**

- Patients with vulvar dermatoses wait an average of 2.9 years until reaching a diagnosis.
- Although cutaneous toxicity is a well-known side effect of immune checkpoint inhibitors (ICIs), onset of inflammatory vulvar dermatoses is delayed compared to other cutaneous adverse events associated with ICI.

**What is new from this article as messages for women and their families?**

- To our knowledge, this presentation of lichenoid tissue eruption resembling erosive lichen planus of the vulva has not previously been reported with ICIs.
- Physicians should be aware of this manifestation that could be underreported, may have late onset, and perhaps have a predilection for women with pre-existing lichen sclerosus.

## Dear Editors,

Cutaneous toxicities are among the most prevalent adverse effects associated with immune checkpoint inhibitors (ICIs) affecting up to 30 to 60% of patients.^1^ We review a case of lichenoid tissue reaction consistent with erosive lichen planus (ELP) of the vulva following pembrolizumab.

A 78-year-old woman presented with a 5-month history of vulvar burning. She denied vaginal discharge or bleeding. Pembrolizumab had been initiated 10 months prior for metastatic endometrial carcinoma.

**Fig. 1.**
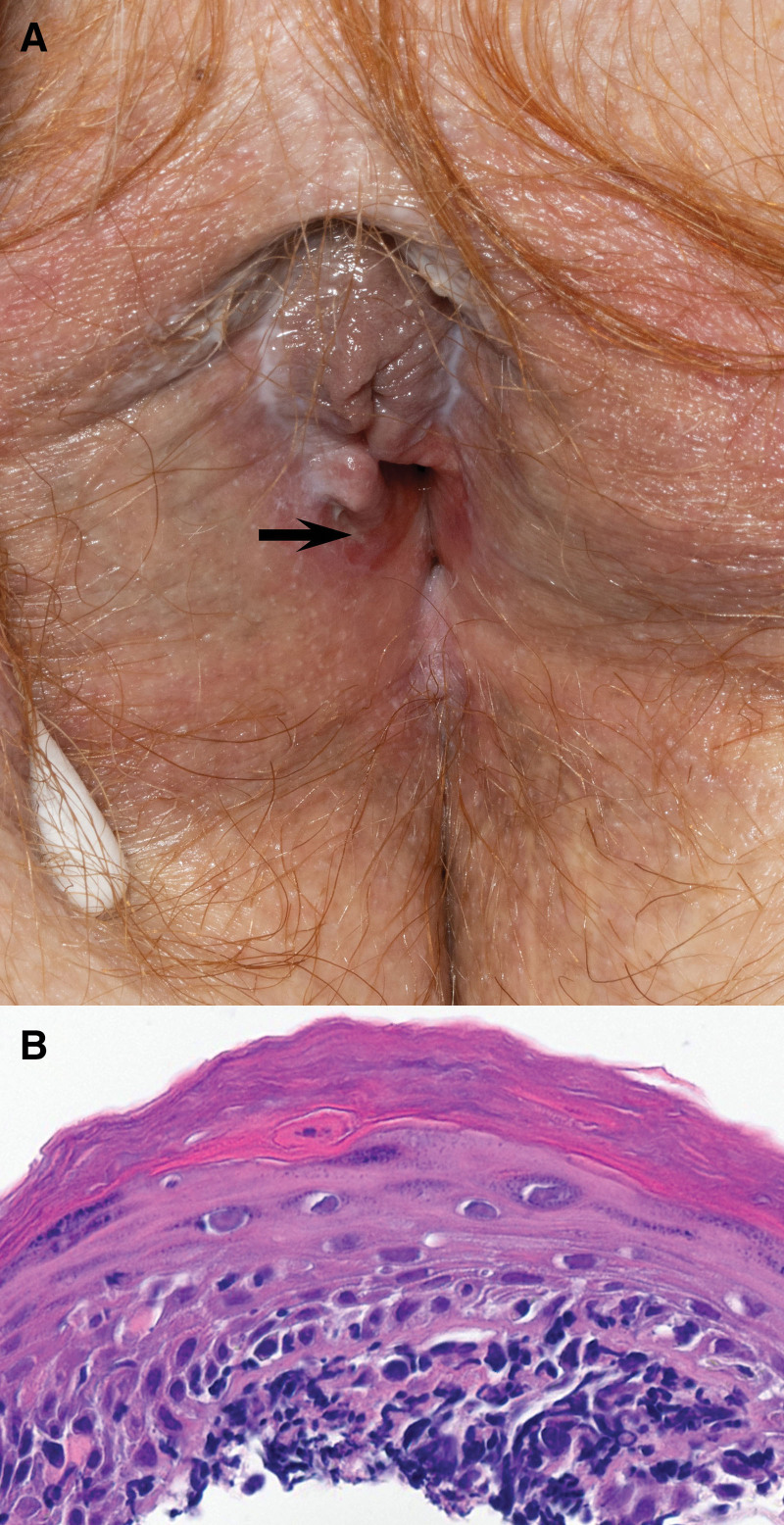
(A) Clinical photo at presentation shows subtle hypopigmentation of clitoral hood and medial labia, partial labial fusion, and whitish to red partially eroded plaques surrounding the vaginal opening within the vestibule medial to Hart’s line. The arrow demonstrates the site of punch biopsy. (B) Histopathology demonstrates dense lymphocytic inflammation at the dermoepidermal junction with associated apoptotic keratinocytes and separation from the underlying dermis (hematoxylin and eosin, ×400).

Physical exam revealed hypopigmentation of the clitoral hood, medial vulva, and labial fusion. The vestibule just medial to Hart’s line was involved by bright red eroded plaques with a white, finely reticulated border (Fig. 1A). Speculum exam was not possible due to discomfort and narrowing of the introitus.

Punch biopsy from the right vestibule near the introitus below Hart’s line showed a dense lichenoid band at the dermal-epidermal junction with many dyskeratotic epidermal keratinocytes, focal subepidermal clefting, and suprabasal acantholysis (Fig. 1B). Follow-up testing evaluated for immunobullous diseases including paraneoplastic pemphigus. Indirect immunofluorescence on salt-split skin, primate esophagus, and rat bladder substrates in addition to enzyme-linked immunosorbent assay serologic antibody testing for desmogleins 1 and 3 and bullous pemphigoid 180 and 230 antibodies were negative. The findings were most consistent with ICI-triggered ELP in the setting of prior lichen sclerosus (LS).

**Fig. 2.**
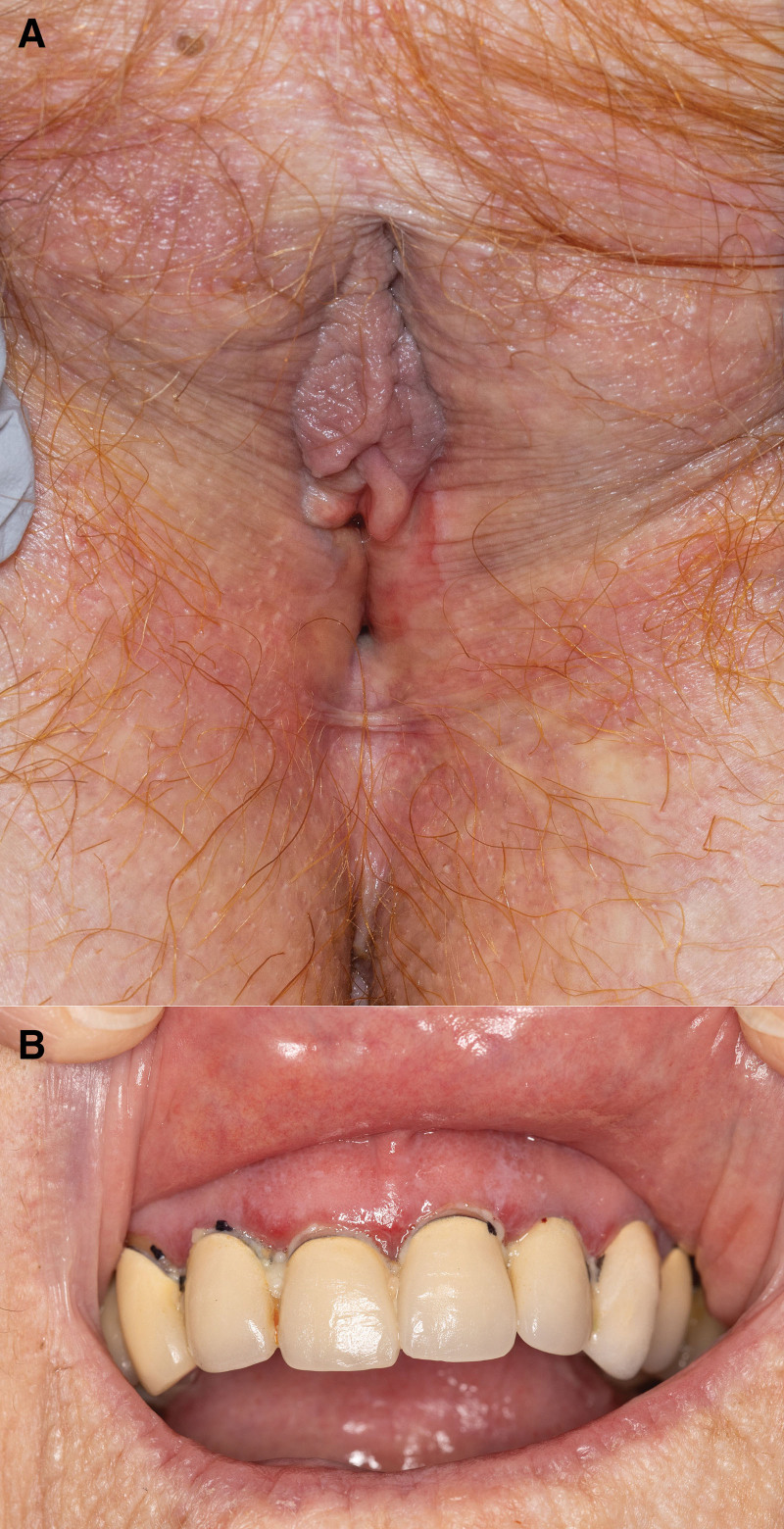
Clinical photos at 12-week follow-up show resolution of red plaques at the right vestibule and decreased erythema at the left vestibule (A) and new erosions at the gingival margin surrounded by fine white recitulated plaques (B).

She initiated clobetasol propionate 0.05% ointment twice daily for 12 weeks. On follow-up, the patient reported relief of burning symptoms, and the vestibular erythema was markedly decreased (Fig. 2A). She endorsed new soreness of the mouth. Erosions at the maxillary gingiva were consistent with desquamative gingivitis associated with oral lichen planus (LP) (Fig. 2B). Treatment with topical clobetasol propionate 0.05% gel was started for oral lesions. Vulvar clobetasol application frequency was tapered to 3 times weekly. At 6-month follow-up, linear pink plaques with focal wickham striae were located on the left ankle.

ELP is an uncommon inflammatory mucocutaneous condition that most commonly affects the oral cavity followed by the genital mucosa with simultaneous involvement in half of patients.^2^ Other less common mucosal manifestations include esophageal, oral, otic, and rarely nasal, laryngeal, anal, and bladder.^3^ ELP is generally considered more refractory to standard treatment with 25 to 40% of patients requiring systemic therapy after failure of topical treatments.^2^ Irreversible scarring is a potential sequela of both vulvar ELP and LS. The risk of vulvar squamous cell carcinoma, although established with LS, is controversial with LP with a recent systematic review not finding a definitive association.^4^

Lichenoid eruptions are a well-reported, delayed reaction in patients receiving ICIs. By removing the inhibition of CTLA-4 and PD-1 on self-reactive T cells, autoimmune disease may arise or worsen during treatment with ICIs.^5^ Inflammatory vulvar dermatoses associated with ICIs, however, are rarely reported. The first genital lichenoid tissue eruption of the vulva associated with ICI was a case of LS reported in 2016.^6^ A recent literature review reported 4 additional cases of genital LS among women aged 50 to 76 treated with ICI.^7^ The median time to onset was 4.6 months. Reported symptoms included dysuria, pruritus, and pain. Patients responded to high-potency topical corticosteroids.^6,7^ A separate publication reported that 76% of patients with cancer treatment-associated vulvar LS were refractory to topical corticosteroids with around 15% necessitating systemic therapy; however, this data was not limited to ICI.^5^ Lichenoid tissue eruptions resembling ELP of the vulva have not previously been reported with ICIs to our knowledge. The current patient’s underlying LS and subsequent development of oral and cutaneous LP suggest that ICI triggers ELP in a predisposed individual.

In summary, dermatologists, gynecologists, and oncologists should be aware of the potential for ICI to trigger new onset and perhaps more importantly flare pre-existing lichenoid eruptions of the vulva. LS is considered a relatively common vulvar disease especially in postmenopausal women and with the expanding use of ICI, this has important implications. Women with LS should be appropriately counseled regarding the risk of disease progression and ELP onset prior to ICI initiation. Prompt diagnosis and treatment are crucial in improving symptoms and diminishing sequela. Prospective studies are needed to further clarify the risk of LS exacerbation and ELP development in patients with underlying LS treated with ICI and to clarify whether ICI-associated lichenoid tissue eruptions of the vulva are more treatment refractory.

## Conflicts of interest

None.

## Funding

None.

## Study approval

N/A

## Author contributions

MID, LAS, and DJD: Participated in the writing of the paper.

## Patient consent

Informed written consent was received from the patient for whom photographs are present in the manuscript.

## Acknowledgments

We thank the patient for permission to publish this case.
